# Antiviral Activity and Possible Mechanism of Action of Constituents Identified in *Paeonia lactiflora* Root toward Human Rhinoviruses

**DOI:** 10.1371/journal.pone.0121629

**Published:** 2015-04-10

**Authors:** Luong Thi My Ngan, Myeong Jin Jang, Min Jung Kwon, Young Joon Ahn

**Affiliations:** 1 Department of Plant Biotechnology and Biotransformation, Faculty of Biology, Ho Chi Minh City University of Science, Vietnam National University, Ho Chi Minh, Vietnam; 2 Biomodulation Major, Department of Agricultural Biotechnology, Seoul National University, Seoul, South Korea; Faculty of Biochemistry Biophysics and Biotechnology, Jagiellonian University, POLAND

## Abstract

Human rhinoviruses (HRVs) are responsible for more than half of all cases of the common cold and cost billions of USD annually in medical visits and missed school and work. An assessment was made of the antiviral activities and mechanisms of action of paeonol (PA) and 1,2,3,4,6-penta-*O*-galloyl-β-D-glucopyranose (PGG) from *Paeonia lactiflora* root toward HRV-2 and HRV-4 in MRC5 cells using a tetrazolium method and real-time quantitative reverse transcription polymerase chain reaction and enzyme-linked immunosorbent assay. Results were compared with those of a reference control ribavirin. Based on 50% inhibitory concentration values, PGG was 13.4 and 18.0 times more active toward HRV-2 (17.89 μM) and HRV-4 (17.33 μM) in MRC5 cells, respectively, than ribavirin. The constituents had relatively high selective index values (3.3–>8.5). The 100 μg/mL PA and 20 μg/mL PGG did not interact with the HRV-4 particles. These constituents inhibited HRV-4 infection only when they were added during the virus inoculation (0 h), the adsorption period of HRVs, but not after 1 h or later. Moreover, the RNA replication levels of HRVs were remarkably reduced in the MRC5 cultures treated with these constituents. These findings suggest that PGG and PA may block or reduce the entry of the viruses into the cells to protect the cells from the virus destruction and abate virus replication, which may play an important role in interfering with expressions of rhinovirus receptors (intercellular adhesion molecule-1 and low-density lipoprotein receptor), inflammatory cytokines (interleukin (IL)-6, IL-8, tumor necrosis factor, interferon beta, and IL-1β), and Toll-like receptor, which resulted in diminishing symptoms induced by HRV. Global efforts to reduce the level of synthetic drugs justify further studies on *P*. *lactiflora* root-derived materials as potential anti-HRV products or lead molecules for the prevention or treatment of HRV.

## Introduction

Human rhinoviruses (HRVs) (Picornaviridae) are the most common cause of upper respiratory tract infection (or common cold) and are responsible for more than half of all cases of the common cold [1,2]. They are also associated with more severe diseases such as acute otitis media in children [3] and sinusitis in adults [4]. HRVs can also cause severe lower respiratory tract symptoms such as pneumonia, wheezing, bronchiolitis, and exacerbations of asthma and chronic obstructive pulmonary disease in infants and children as well as fatal pneumonia in elderly and immunocompromised adults [1,2]. Although HRV-induced upper respiratory illness is often mild and self-limiting, the socioeconomic burden caused by medical visits and missed school and work by HRV infection is enormous [2,5,6]. The degree of drug misuse and abuse is significant and antihistamine and antibiotic usages have caused many side effects [7]. Attempts to develop effective treatments or vaccination have been relatively limited and unsuccessful because of more than 100 serotypes of HRV [1,2,8]. There is a need for the development of selective antiviral agents with novel target sites to establish an effective HRV management strategy and tactics because currently no effective antiviral therapies have been approved for either the prevention or treatment of diseases caused by HRV infection [2].

Plants may provide potential sources of antiviral products largely because they constitute a potential source of bioactive secondary metabolites that have been perceived by the general public as relatively safe, with minimal impacts to human health, and often act at multiple and novel target sites [9–12]. Certain plant preparations and their constituents are regarded as potential sources for commercial antiviral products for prevention or treatment of HRV infection. Previous studies have shown that a methanol extract from the root of Chinese peony, *Paeonia lactiflora* Pallas (Paeoniaceae), possessed good antiviral activity toward HRV-2 and HRV-4. No work has been obtained concerning the potential use of *P*. *lactiflora* to manage HRV, although historically *P*. *lactiflora* root (2–4 g of dried root/3 times/day) is used as analgesic, hemostyptic, and bacteriostatic agents [13,14].

The aim of the study was to assess the cytotoxic and antiviral effects on two cell lines (HeLa and MRC5) and two HRV serotypes (HRV-2 and HRV-4) of paeonol (PA), gallic acid (GA), and 1,2,3,4,6-penta-*O*-galloyl-β-D-glucopyranose (PGG) from *P*. *lactiflora* root using a 3-(4,5-dimethylthiazol-2-yl)-2,5-diphenyl tetrazolium bromide (MTT) assay. The antiviral activities of these materials were compared with those of ribavirin, a broad-spectrum antiviral agent currently used clinically to treat various DNA and RNA virus infections [15]. The antiviral properties and mechanisms of action of the constituents also were elucidated using real-time quantitative reverse transcription polymerase chain reaction (qRT-PCR) with SYBR Green dye and specific enzyme-linked immunosorbent assay (ELISA).

## Materials and Methods

### Instrumental analysis

^1^H and ^13^C NMR spectra were recorded in CD_3_OD on an AVANCE 600 spectrometer (Bruker, Rheinspettem, Germany) at 600 and 150 MHz, respectively, using tetramethylsilane as an internal standard, and chemical shifts are given in δ (ppm). UV spectra were obtained in methanol on a UVICON 933/934 spectrophotometer (Kontron, Milan, Italy), mass spectra on GMS-600 W or JMS-700 spectrometer (Jeol, Tokyo, Japan), and FT-IR spectra on a Nicolet Magna 550 series II spectrometer (Midac, Irvine, CA). Optical rotation was measured with an Autopol III polarimeter (Rudolph Research Analytical, Flanders, NJ). Silica gel 60 (0.063–0.2 mm) (Merck, Darmstadt, Germany) was used for column chromatography. Merck precoated silica gel plates (Kieselgel 60 F_254_) were used for analytical thin-layer chromatography (TLC). Merck silica gel 60 RP-18 F_254S_ plates (for RP-TLC) and an Agilent 1200 series high-performance liquid chromatograph (HPLC) (Santa Clara, CA) were used for isolation of active principles.

### Materials

Ribavirin (>98% purity) and MTT were purchased from Tokyo Chemical Industry (Tokyo, Japan) and Sigma-Aldrich (St. Louis, MO), respectively. Anitbiotic-antimycotic and minimum essential medium (MEM) were purchased from Invitrogen (Grand Island, NY). Fetal bovine serum (FBS) was supplied by PAA Laboratories (Etobicoke, Ontario, Canada). The protein molecular weight standards (Precision Plus Protein all blue standards) were purchased from Bio-Rad Life Sciences (Hercules, CA). RIPA buffer and 1% mammalian cell protease inhibitor cocktail were purchased from Sigma-Aldrich. The primary antibodies used in this study were as follows: anti-ICAM-1 antibody [rabbit polyclonal to intercellular adhesion molecule-1 (ICAM-1)], anti-low-density lipoprotein receptor (LDLR) antibody (rabbit polyclonal to LDLR), and anti-actin antibody (rabbit polyclonal to actin) purchased from Abcam (Cambridge, UK). The secondary antibody (horseradish peroxidase conjugated goat polyclonal to rabbit) was supplied by Abcam. All of the other chemicals and reagents used in this study were of analytical grade quality and available commercially.

### Cell lines and human rhinovirus serotypes

HeLa (ATCC CCL–2), a human epithelial adenocarcinoma cervix cell line, and MRC5 (ATCC CCl-171), a human lung fibroblast cell line, were purchased from the American Type Culture Collection (ATCC) (Manassas, VA). These cell lines were maintained in MEM supplemented with 10% FBS and antibiotic-antimycotic solution (100000 units/L of penicillin, 100 mg/L of streptomycin, and 250 μg/L of amphotericin) in a humidified incubator at 37°C and 5% CO_2_. HRV-2 (ATCC VR-1112AS/GP) and HRV-4 (ATCC VR-1114AS/GP) were purchased from ATCC. Virus titers were determined by cytopathic effects (CPE) in HeLa and MRC5 cells and were expressed as 50% tissue culture infective dose (TCID_50_) per mL as described previously by Morgan [16].

### Bioassay-guided fractionation and isolation

**Fig 1:**
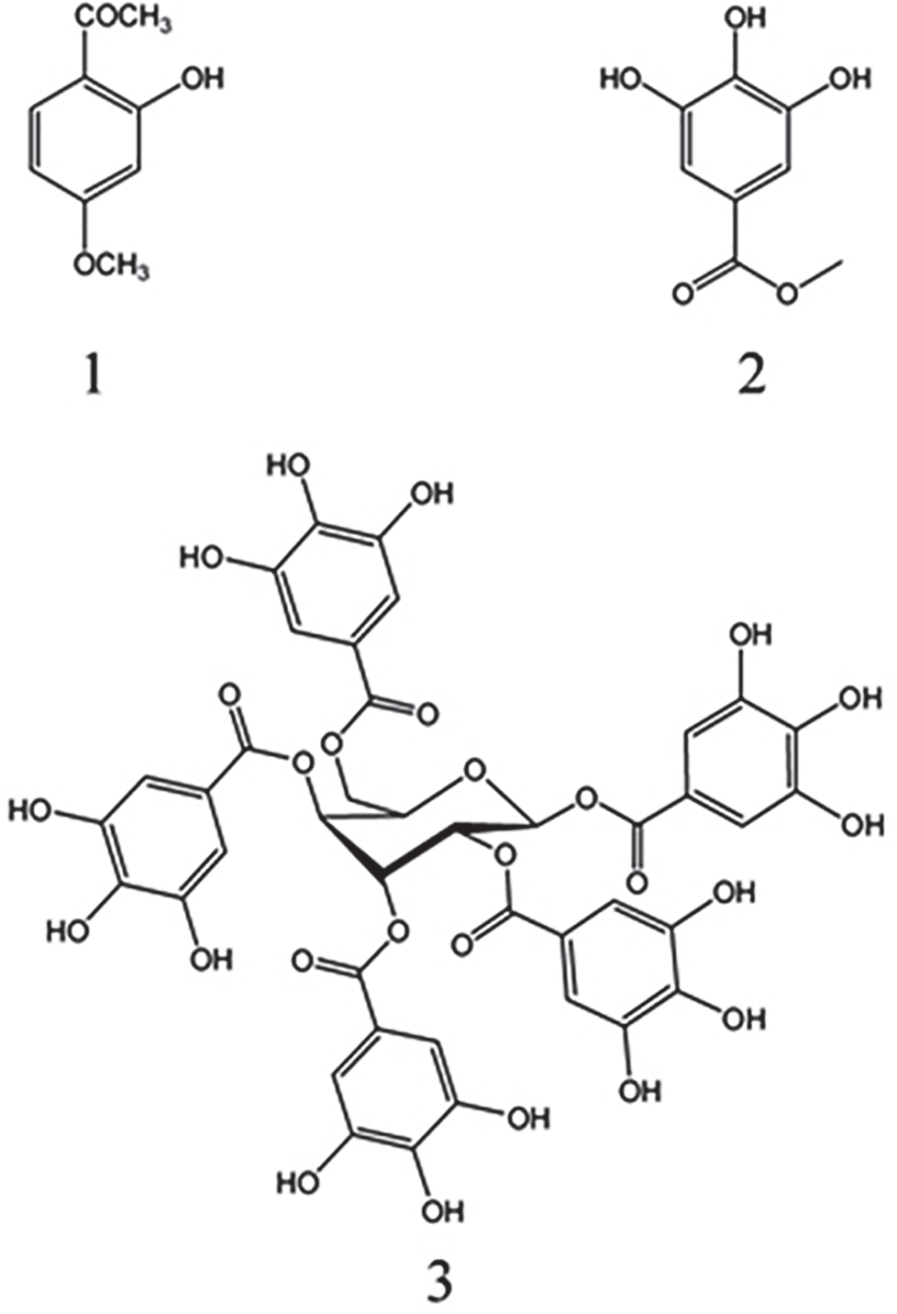
Structures of paeonol (1), gallic acid (2), and 1,2,3,4,6-penta-*O*-galloyl-β-D-glucopyranose (3). These compounds were identified in *Paeonia lactiflora* root in this study. The chemical formulae of these compounds are C_9_H_10_O_3_, C_7_H_6_O_5_, and C_41_H_32_O_26_; the molar masses are 166.18, 170.12, and 940.67 g/mol.

Extraction procedures of air-dried root of *P*. *lactiflora* were performed as described previously by Ngan et al. [17]. For isolation of active principles, viral CPE inhibition assay [18] toward HRV-4 in HeLa cell was used. The hexane-soluble fraction was most biologically active (Table 1) and was chromatographed as described previously [17]. Finally, an active principle **1** (94 mg) was isolated at a retention time of 10.9 min. The other active ethyl acetate-soluble fraction (8 g) was chromatographed on a 55 × 5 cm silica gel column (550 g) by elution with a gradient of chloroform and methanol [100:0 (1 L), 99:1 (1 L), 95:5 (1 L), 90:10 (2 L), 80:20 (1 L), 70:30 (1 L), and 0:100 (1 L) by volume] to provide eight fractions (each about 1 L). Column fractions were monitored by TLC on silica gel plates developed with chloroform and methanol (7:3 by volume) mobile phase. Fractions with similar *R*_f_ values on the TLC plates were pooled. Active fractions 4 to 5 (EI, 2.44 g) and 7 (EII, 0.569 g) were obtained. The active fraction EI was rechromatographed on a silica gel column by elution with chloroform and methanol (70:30 by volume) to give nine fractions (each about 450 mL). A preparative HPLC was used for separation of constituents from the active fractions 3 to 5 (0.176 g). The column was a 4.6 mm i.d. × 150 mm Eclipse XDB-C_18_ (Agilent, Santa Clara, CA) using a mobile phase of methanol and water (3:7 by volume) at a flow rate of 0.5 mL/min. Chromatographic separations were monitored using a UV detector at 260 nm. Finally, an active principle **2** (75 mg) was isolated at a retention time of 4.83 min. Fraction EII was purified by RP-TLC with chloroform:methanol:water (70:25:5 by volume) to afford an active principle **3** (*R*_f_ = 0.72, 45 mg). The three antiviral principles were characterized as paeonol (PA) (**1**), gallic acid (GA) (**2**), and 1,2,3,4,6-penta-*O*-galloyl-β-D-glucopyranose (PGG) (**3**) (Fig 1) by spectroscopic analyses, including MS and NMR. PA (**1**): compound **1** was isolated as an antibacterial principle from *P*. *lactiflora* root in our previous work [17], and the spectral data of compound **1** was largely identical to the published data [17]. GA (**2**) was identified on the basis of the following evidence: white powder. UV (MeOH): λ_max_ nm = 260. FT-IR: *v*_max_ cm^–1^ = 3491–3063 (OH stretch), 1608 (aromatic C = C). EI-MS (70eV), *m/z* (% relative intensity): 170.1 [M]^+^ (100), 153 (66.1), 125 (11.6), 124 (3), 107 (3.2), 79 (6.5), 78 (2.1). ^1^H NMR (CD_3_OD, 600 MHz): δ 7.03 (2H, s). ^13^C NMR (CD_3_OD, 150 MHz): δ 110.4 d, 122.1 s, 139.7 s, 146.5 s, 170.6 s. The interpretations of proton and carbon signals of compound **2** were largely consistent with those of Sakar et al. [19]. PGG (**3**): compound **3** was isolated as an antibacterial principle from *P*. *lactiflora* root in our previous work [17], and the spectral data of compound **3** was largely identical to the published data [17,20].

**Table 1:** Cytoxicity and antiviral activity of fractions obtained from the solvent hydrolyzable of the methanol extract of *Paeonia lactiflora* root toward human rhinovirus-4 in HeLa cells using a tetrazolium assay.

Test material	CC_50_ (μg/mL) (95% CL)	IC_50_ (μg/mL) (95% CL)	SI
**Methanol extract**	>1000	113.5 (102.8–125.4)	>10
**Haxane-SF**	254.0 (242.3–266.3)	70.2 (66.7–73.8)	3.6
**Chloroform-SF**	136.0 (125.4–157.6)	ND	
**Ethyl acetate-SF**	552.9 (504.1–606.5)	121.5 (117.1–125.9)	4.6
**Butanol-SF**	>1000	>1000	
**Water-SF**	>1000	>1000	

CC_50_, 50% cytotoxic concentration; IC_50_, 50% inhibitory concentration; SI, selectivity index; SF, soluble fraction; ND, no determination.

### Cytotoxicity assay

The cytotoxicity of the test materials to two human cell lines was evaluated using an MTT assay described previously by Morgan [16]. In brief, a 10× stock solution of MTT was prepared by adding 5 mg/mL MTT in phosphate-buffered saline (PBS) (pH 7.4). The stock solution was sterile-filtered and stored at −20°C. HeLa and MRC5 cells were seeded onto 96-well culture plates at a density of 3 × 10^4^ cells per well for 1 day. The culture medium was removed and the plates with monolayer cells were replaced with media containing several different concentrations (1–1000 μg/mL) of the test materials in dimethylsulfoxide (DMSO). After incubation at 37°C and 5% CO_2_ for 2 days, the culture plates were then washed once with 200 μL PBS. A volume of 100 μL medium containing 0.05% MTT were added to each well and then incubated for 4 h at the same condition. After then, MTT solution was removed and 150 μL DMSO was added to each well. Finally, the plate was shaken for 15 min to dissolve the purple formazan crystals that had formed. Absorbance was read at 560 nm by using a VersaMax microplate reader (Molecular Devices, Sunnyvale, CA) with a reference absorbance at 670 nm.

### Cytopathic effect inhibition assay

Monolayers of HeLa and MRC5 cells were seeded onto a 96-well culture plate as stated previously. Subsequently, 90 μL media containing several different concentrations (1–1000 μg/mL) of each test material in DMSO was put into the wells, and then 10 μL of 10×TCID_50_ of the virus stock was added to produce an appropriate CPE within 2 days after infection. Ribavirin served as a reference control and was similarly prepared. Negative controls consisted of the DMSO solution. Viral inhibition rate (VIR) (%) was calculated according to the formula [21], VIR = (*A*_tV_—*A*_cV_)/(*A*_cd_—*A*_cV_) × 100, where *A*_tV_ is the optical density measured with a given concentration of the test compound in HRV infected cells; *A*_cV_ is the optical density measured for the control untreated HRV infected cells; *A*_cd_ is the optical density measured for the control untreated HRV uninfected cells.

### Virus titration assay

Virus titers in infected MRC-5 cultures treated with PA and PGG were measured as TCID_50_ using MTT-based titration method [22]. In brief, MRC5 cells were seeded at 10^5^ cells/mL in a 6-well plate. After 24 h, the cell monolayers were treated with 100 μg/mL PA or 20 μg/mL PGG and infected with HRV-2 or HRV-4 in a concentration of 10×TCID_50_/mL. Uninfected untreated and infected untreated cultures were included in the assay. The 100 μg/mL of ribavirin was used as a reference control. After 48 h incubation at 37°C, the cultures were frozen and thawed at −80°C/25°C. Cell debris was removed by centrifugation (2000 rpm) and the virus supernatants were collected. Virus titration was then performed in HeLa cell cultures. Initially, a 1:10 dilution of each supernatant was prepared followed by 10 serial 2-fold-dilutions, and added to HeLa cell monolayers. After 48 h, cell mortality was measured using an MTT assay stated previously. Graphs were built by plotting dead cell percentages toward virus dilution factors of each virus supernatant. The 50% infectivity point was calculated through a linear regression analysis of the curve.

### Infectivity of human rhinovirus particles

The effects of the test compounds on the infectivity of HRV-4 particles were elucidated as described previously by Choi et al. [23]. Approximately 1.5-fold quantities of the IC_50_ values of each test compound were applied. HRV-4 was preincubated with 100 μg/mL PA, 20 μg/mL PGG, or 100 μg/mL ribavirin for 1 h at 4°C. Monolayers of MRC5 cells were infected with the pretreated or untreated HRV-4 for 1 h at 37°C. Unbound virus was removed by washing the wells with PBS twice, and then cells were incubated in fresh medium supplemented with or without test compounds at 37°C. After 2 days, MTT test and antiviral activity were carried out as stated previously.

### Time course

The time-of-addition effects of all compounds on HRV-4 were examined according to the method of Choi et al. [23]. In brief, monolayers of MRC5 cells were seeded onto a 96-well culture plate as stated previously. After washing with PBS, 100 μg/mL PA, 20 μg/mL PGG, and 100 μg/mL ribavirin were separately added onto the cells at either before (–1 h), during (0 h), or after (1, 2, 3, 4, 6, 8, 12, 16, 20, and 24 h) HRV-4 infection at 37°C. After 2 days, MTT test and antiviral activity were carried out as stated previously.

### Real-time reverse transcription-PCR analysis

To evaluate the level of gene expression, real-time qRT-PCR with SYBR Green dye was carried out. HRV-2 or HRV-4 infected and noninfected cultures of MRC5 monolayers grown in 25 cm^2^ cell culture flasks (Corning, NY) were treated with 100 μg/mL PA or 20 μg/mL PGG. After incubation at 37°C and 5% CO_2_ for 2 days, total RNA was extracted from the culture cells using the RNeasy Plus Mini Kit (Qiagen, Hilden, Germany) according to the manufacturer’s instructions. Contaminated genomic DNA was removed using RQ1 RNase-free DNase (Promega, Madison, WI). Complementary DNA (cDNA) was synthesized using 1 μg total RNA through a reverse transcription reaction using the SuperScript First-Strand Synthesis Kit (Invitrogen, Carlsbad, CA). Five log10-fold dilutions of cDNA for each RNA were performed to determine PCR efficiency (100 ng–10 pg per reaction). qRT-PCR was performed in 96-well plates using the StepOnePlus Real-Time PCR System (Applied Biosystems, Foster, CA). Each reaction mixture consisted of 10 μL of Maxima SYBR Green/ROX qPCR Master Mix (2×) (Thermo Scientific, Foster, CA), 2 μL of forward and reverse primers (5 pmol each), 1 μL of cDNA (8 ng), and 7 μL of double-distilled water in a final volume of 20 μL. Oligonucleotide PCR primer pairs are listed in Table 2 and were purchased from Applied Biosystems. The PCR conditions were as follows: 50°C for 2 min, 95°C for 10 min, and then 50 cycles of 95°C for 15 s and either 60°C [β2-microglobulin (B2M), interleukin (IL)-6, IL-8, LDLR, ICAM-1, HRV-2, and HRV-4] or 58°C (B2M and IL-1β) or 55°C [B2M, Toll-like receptor 3 (TLR3), tumor necrosis factor (TNF), and interferon beta (IFNβ)] for 30 s. mRNA expression level of target gene was normalized to mRNA expression level for the housekeeping gene B2M and analyzed by the 2^–ΔΔ*C*T^ method using StepOne Software v2.1 and DataAssist Software (Applied Biosystems). The RNA expression of B2M was not different in HRV-infected MRC-5 cultures and mock cultures. Therefore, it was used as an internal standard for virus replication and cytokine mRNA expression.

**Table 2:** Primers used for real-time quantitative reverse transcription polymerase chain reaction in this study.

Gene	RefSeq ID	Forward primer and Reverse primer	cDNA amplicon size
**B2M**	AF072097	CTCCGTGGCCTTAGCTGTG TTTGGAGTACGCTGGATAGCCT	68
**ICAM-1**	NM_000201.2	ACCTCCCCACCCACATACATTT GGCATAGCTTGGGCATATTCC	96
**LDLR**	NM_000527	CTGGAAATTGCGCTGGAC CGCAGACCCACTTGTAGGAG	125
**IL-6**	M14584	GACCCAACCACAAATGCCA GTCATGTCCTGCAGCCACTG	68
**IL-8**	NM_000584	CTGGCCGTGGCTCTCTTG CCTTGGCAAAACTGCACCTT	69
**TLR3**	NM_003265	TCCCAAGCCTTCAACGACTG TGGTGAAGGAGAGCTATCCACA	68
**TNF**	M10988	GGTGCTTGTTCCTCAGCCTC CAGGCAGAAGAGCGTGGTG	65
**IL-1β**	M15330	ACGAATCTCCGACCACCACT CCATGGCCACAACAACTGAC	65
**IFNβ**	M28622	CAGCAATTTTCAGTGTCAGAAGC TCATCCTGTCCTTGAGGCAGT	74
**HRV- 4**	DQ473490.1	CGGCCCCTGAATGCGGCTAA GAAACACGGACACCCAAAGTA	115
**HRV- 2**	X02316.1	CGGCCCCTGAATGTGGCTAA GAAACACGGACACCCAAAGTA	115

### Western blot analysis

Cell lysates from infected and noninfected MRC5 cultures 2 days after incubation with or without test compounds were obtained in RIPA buffer and 1% mammalian cell protease inhibitor cocktail according to the manufacturer’s instructions. Working dilutions of primary antibodies were dilulted 500, 2000, and 1000 times for anti-ICAM-1, anti-LDLR, and anti-actin antibodies, respectively. Working dilutions of secondary antibody was 1000, 1000, and 800 for anti-ICAM-1, anti-LDLR, and anti-actin, respectively. Ten micrograms of cell lysates from different treatments were mixed with an equal volume of 5× Laemmli sample buffer, boiled in 10 min, and resolved by electrophoresis in 11% sodium dodecyl sulfate-polyacrylamide gels (SDS-PAGE) [24]. After electrophoresis at 120 V in 2 h, proteins from the gels were transferred onto a polyvinyl difluoride membrane (Pall Corporation, Pensacola, FL) using a electroblotting apparatus (Bio-Rad, Hercules, CA). The membranes for anti-ICAM-1 and anti-LDLR were incubated in blocking solution containing 5% nonfat dry milk for 4 h to inhibit nonspecific binding. These membranes were then incubated overnight at 4°C with the primary antibodies. After washing with PBS (each 10 min) three times, the membranes were further incubated with the secondary antibody for 2 h and washed with PBS containing 0.5% Tween-20 (v/v) (0.5% PBS-T) four times (each 15 min). The membranes for anti-actin were incubated in PBS blocking solution containing 5% BSA overnight to inhibit nonspecific binding, and then incubated with anti-actin antibody in the blocking solution for 3 h at 25°C and washed four times (each 15 min) in 0.1% PBS-T at room temperature before incubation with the second antibody. Finally, all the membranes were developed with an ECL chemiluminescence reagent (Amersham Bioscience, Buckinghamshire, UK) and exposed to a CP-PU X-ray film (AGFA, Mortsel, Belgium). Differences in protein expressions were quantified using a Molecular Imager Gel Doc XR system (Bio-Rad, Hercules, CA) and normalized to actin expression on the same membrane.

### Measurement of ICAM-1 and LDLR expression

The expressions of ICAM-1 and LDLR were examined using real-time qRT-PCR and Western blot. Furthermore, concentrations of soluble ICAM-1 (sICAM-1) in cell-free culture supernatants 2 days after treatment were measured using a sICAM-1 ELISA Kit (Pierce Biotechnology, Rockford, IL) according to the manufacturer’s instructions. The sensitivities of the assays were 0.3 ng/mL. The concentrations of ICAM-1 in the test samples were determined from OD values using standard curve of each assay.

### Measurement of cytokine production

The concentrations of IL-6, IL-8, and TNF in cell-free culture supernatants 2 days after treatment were measured using specific ELISA. OptEIA IL-6, IL-8, and TNF ELISA kits (BD Biosciences, San Diego, CA) were used for the assays. The sensitivities of these ELISA assays were 2.2, 0.8, and 2.0 pg/mL, respectively. The concentrations of IL-6, IL-8, and TNF in the test samples were determined from OD values using standard curve of each assay.

### Data analysis

Cytotoxicity was expressed as 50% cytotoxic concentration (CC_50_) of each compound that reduced the viability of cells to 50% of the control. Fifty percent inhibitory concentration (IC_50_) was defined as the compound concentration required to reducing the viral CPE to 50% of the control. The CC_50_ and IC_50_ values were determined using GraphPad Prism 5 software program (GraphPad Software, La Jolla, CA). The IC_50_ values for each serotype and their treatments were considered to be significantly different from one another when their 95% confidence limits (CLs) did not overlap. The selectivity index (SI) was determined as the ratio of CC_50_ to IC_50_ [23]. All data represent the mean ± SD of duplicate or triplicate samples of three independent experiments. Statistical analyses were carried out using SAS 9.13 program (SAS Institute, Cary, NC). Data from two groups were analyzed by a Student’s *t*-test, and multiple groups were analyzed by a one-way analysis of variance and Bonferroni multiple comparison post-test.

## Results

### Anti-human rhinovirus activity of test compounds

The antiviral activity of PGG, GA, and PA toward HRV-2 and HRV-4 in HeLa cells was compared with that of ribavirin using an MTT assay (Table 3). Based on IC_50_ values, PGG was the most active constituent toward HRV-2 (11.56 μM) and HRV-4 (14.38 μM) and was 26.3 and 22.5 times more active than ribavirin (303.56 and 324.07 μM), respectively. The antiviral activity of GA (IC_50_, 426.99 μM for HRV-2; 448.10 μM for HRV-4) and ribavirin did not differ significantly. The antiviral activity of PA (IC_50_, 608. 38 μM for HRV-2; 513.84 μM for HRV-4) was the lowest of any of the test compounds. These compounds were not cytotoxic toward HeLa cells (CC_50_, 108.8–3159.2 μM).

**Table 3:** Cytoxicity and antiviral activity of test compounds and antiviral agent ribavirin toward human rhinovirus-2 and -4 in HeLa cells using a tetrazolium assay.

Compound^a^	CC_50_ (μM) (95% CL)	IC_50_ (μM) (95% CL)		SI	
		HRV-2	HRV-4	HRV-2	HRV-4
**PGG**	108.8 (98.0–120.7)	11.6 (9.8–13.7)	14.4 (12.3–16.8)	9.4	7.6
**GA**	1500.1 (1303.2–1727.0)	427.0 (386.3–472.0)	448.1 (407.5–492.8)	3.5	3.3
**PA**	3159.2 (2921.0–3397.3)	608.4 (575.9–643.3)	513.8 (473.5–557.6)	5.2	6.1
**Ribavirin**	2341.1 (2151.1–2534.2)	303.6 (276.3–333.4)	324.1 (294.4–356.7)	7.7	7.2

CC_50_, 50% cytotoxic concentration; IC_50_, 50% inhibitory concentration; SI, selectivity index; HRV, human rhinovirus.

^a^PGG, 1,2,3,4,6-penta-*O*-galloyl-β-D-glucopyranose; GA, gallic acid; PA, paeonol.

The antiviral effects of all compounds on HRV-2 and HRV-4 in MRC5 cells was likewise evaluated (Table 4). As judged by IC_50_ values, PGG was the most active constituent toward HRV-2 (17.89 μM) and HRV-4 (17.33 μM) and was 13.4 and 18.0 times more active than ribavirin (240.49 and 311.70 μM), respectively. PA (IC_50_, 503.13 μM) exhibited significantly lower antiviral activity than ribavirin toward HRV-2. The antiviral activity of PA (IC_50_, 492.17 μM) and ribavirin toward HRV-4 did not differ significantly. GA was ineffective toward both virus serotypes at all test concentrations which were less than or equal to its CC_50_ (217.5 μM). At concentrations CC ≥290 μM, GA was toxic to cells. PGG, PA, and ribavirin were not cytotoxic toward MRC5 (CC_50_, 108.4–3144.2 μM).

**Table 4:** Cytoxicity and antiviral activity of test compounds and antiviral agent ribavirin toward human rhinovirus-2 and -4 in MRC5 cells using a tetrazolium assay.

Compound^a^	CC_50_ (μM) (95% CL)	IC_50_ (μM) (95% CL)		SI	
		HRV-2	HRV-4	HRV-2	HRV-4
**PGG**	108.4 (101.7–115.5)	17.9 (15.9–20.2)	17.3 (14.7–20.4)	6.1	6.3
**GA**	217.5 (188.3–251.2)	ND	ND		
**PA**	3144.2 (2863.2–3452.3)	503.1 (420.1–602.4)	492.2 (436.4–555.1)	6.2	6.4
**Ribavirin**	2229.7 (2053.2–2421.7)	240.5 (208.3–277.7)	311.7 (292.4–332.3)	9.1	7

CC_50_, 50% cytotoxic concentration; IC_50_, 50% inhibitory concentration; SI, selectivity index; HRV, human rhinovirus; ND, no determination.

^a^PGG, 1,2,3,4,6-penta-*O*-galloyl-β-D-glucopyranose; GA, gallic acid; PA, paeonol.

### Effect on virus titers

Effects of PA and PGG on HRV titers were compared with those of ribavirin (Table 5). Treatment with 100 μg/mL PA and 20 μg/mL PGG resulted in reducing HRV-2 replication by log0.88 and log0.68, or reducing the HRV-2 titer by 22.4 and 17.3%, respectively. Similarly, the HRV-4 titers were reduced in the cultures treated with PA and PGG by 27.6 and 20.7%, respectively. Treatment with 100 μg/mL ribavirin resulted in reducing HRV-2 and HRV-4 titers by 30.3 and 36.0%, respectively.

**Table 5:** Effects of test compounds on virus titer.

Compound^a^	Log (TCID_50_/mL) ± SE		Reduction of virus replication (Log (TCID_50_/mL)) ± SE		
	HRV-2	HRV-4	HRV-2	HRV-4	*P*-value^c^
**PGG 20 μg/mL**	3.25 ± 0.075 b^b^	3.04 ± 0.038 c	0.68 ± 0.075	0.79 ± 0.038	0.2591
**PA 100 μg/mL**	3.05 ± 0.046 b	2.76 ± 0.024 b	0.88 ± 0.046	1.05 ± 0.023	0.0158
**Ribavirin 100 μg/mL**	2.74 ± 0.020 a	2.44 ± 0.024 a	1.19 ± 0.020	1.37 ± 0.023	0.0008
**Control (infected untreated)**	3.93 ± 0.032 c	3.81 ± 0.043 d			

TCID_50_, 50% tissue culture infective dose; HRV, human rhinovirus.

^a^PGG, 1,2,3,4,6-penta-*O*-galloyl-β-D-glucopyranose; PA, paeonol.

^b^Bonferroni multiple comparison post-test (*p* = 0.05).

^c^Student *t*-test.

### Effect on the infectivity of human rhinovirus particles

**Fig 2:**
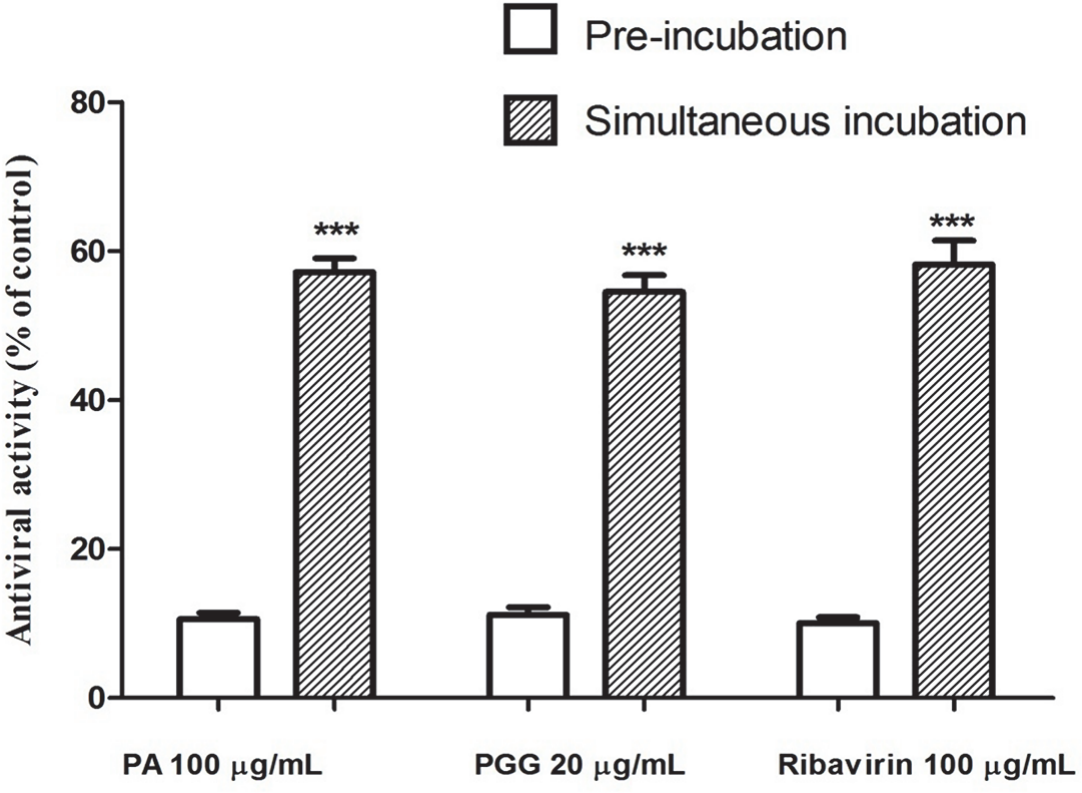
Effect on the infectivity of HRV-4 particles. Human rhinovirus-4 (HRV-4) particles were incubated with 100 μg/mL paeonol (PA), 20 μg/mL 1,2,3,4,6-penta-*O*-galloyl-β-D-glucopyranose (PGG), and 100 μg/mL ribavirin for 1 h at 4°C. Afterwards, MRC5 cells were incubated with treated or untreated virus for 1 h at 37°C. Unbound viruses were removed and washed by phosphate-buffered saline twice, and then cells were incubated in fresh medium with or without test compounds. After 2 days, inhibition was evaluated by tetrazolium method and expressed as the inhibition rate. Each bar represents the mean ± SD of triplicate samples of three independent experiments. ***p<0.001, using a Student’s *t*-test.

Due to their antiviral activity with high selectivity, the effects of PA and PGG on the infectivity of HRV-4 particles were likewise compared with those of ribavirin (Fig 2). The inhibition rates of preincubation with 100 μg/mL PA, 20 μg/mL PGG, and 100 μg/mL ribavirin were 10.6, 11.2, and 10.0%, respectively. Continuous presence of PA, PGG, and ribavirin during infection led to a significant increase in the inhibition rate (57.2, 54.5, and 58.2%).

### Time course of compound addition

**Fig 3:**
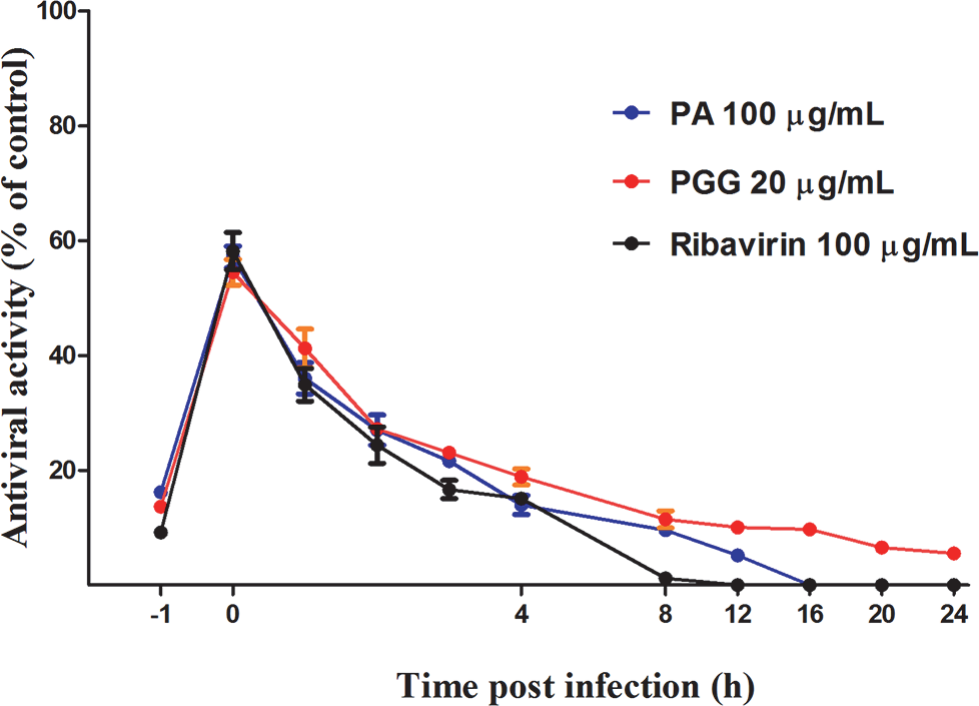
Time-of-addition effect on HRV-4 replication in MRC5 cells. The 100 μg/mL paeonol (PA), 20 μg/mL 1,2,3,4,6-penta-*O*-galloyl-β-D-glucopyranose (PGG), and 100 μg/mL ribavirin were added at various times preinfection (–1 h), coinfection (0 h), or postinfection (1, 2, 4, 6, 8, 12, 16, 20, and 24 h) of human rhinovirus-4 (HRV-4). After 2 days, inhibition was evaluated by tetrazoilum method and expressed as the inhibition rate. Each bar represents the mean ± SD of triplicate samples of three independent experiments.

To investigate the mode of action of PA, PGG, and ribavirin, time-of-addition experiments were performed (Fig 3). Treatment with 100 μg/mL PA or 20 μg/mL PGG considerably suppressed HRV-4 infection only when added just after the virus inoculation (0 h) (57.2 and 54.5% inhibition). The inhibition of these compounds declined to 40% or less when added at either prior (–1 h) or post (1–24 h) infection. Similar results were observed with 100 μg/mL ribavirin.

### Effect on the level of human rhinovirus replication

**Fig 4:**
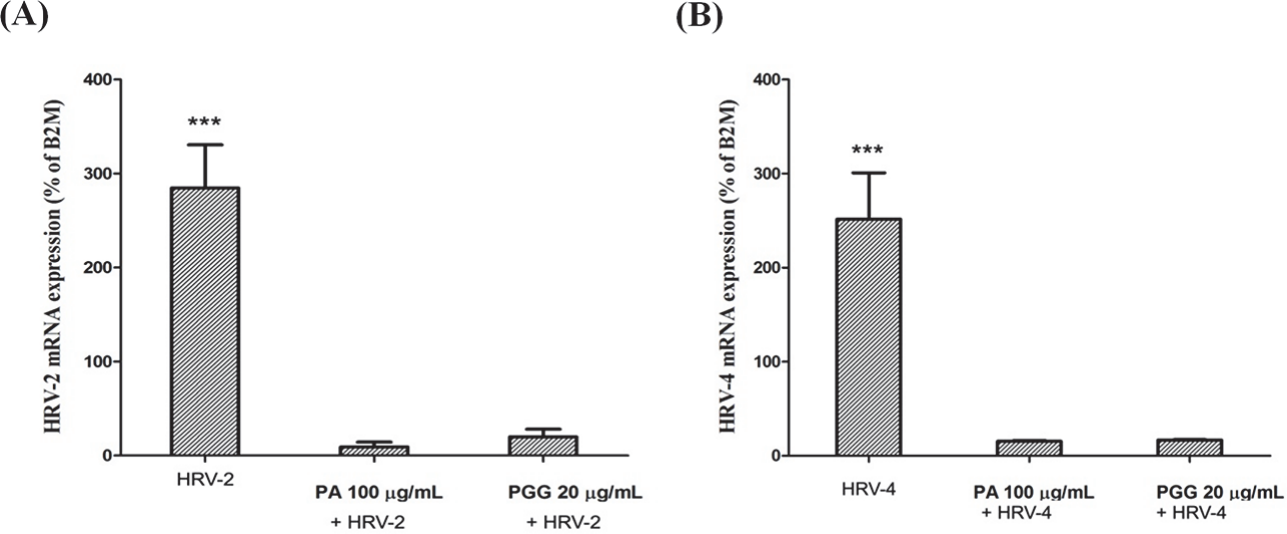
Effect on the level of HRV replication. The RNA replication levels of human rhinovirus-2 (HRV-2) (**A**) and HRV-4 (**B**) were detected by real-time quantitative reverse transcription-PCR with SYBR Green dye in MRC5 cells 2 days after infection in the presence of 100 μg/mL paeonol (PA) and 20 μg/mL 1,2,3,4,6-penta-*O*-galloyl-β-D-glucopyranose (PGG). HRV mRNA expressions were normalized to the constitutive expression of mRNA of the housekeeping gene β2-microglobulin (B2M) and analyzed by the 2^−ΔΔ*C*T^ method using StepOne Software v2.1 and DataAssist Software. Each bar represents the mean ± SD of duplicate samples of three independent experiments. ***p<0.001, using a Bonferroni multiple comparison post-test.

Further evidence of the inhibitory effects of PA and PGG on HRV replication in MRC-5 cells was provided by real-time qRT-PCR analysis (Fig 4). In the presence of 100 μg/mL PA or 20 μg/mL PGG in MRC5 cell cultures infected with HRV-2, the RNA replication levels were reduced by 30.1 and 14.3 fold, respectively, compared to the levels in the cell cultures without the compounds (Fig 4A). Similarly, the replication levels of the HRV-4 in the MRC5 cell culture treated with PA or PGG were also reduced by 16.3 and 15.1 fold, respectively, compared with the untreated cultures (Fig 4B).

### Effect on ICAM-1 and LDLR expressions

**Fig 5:**
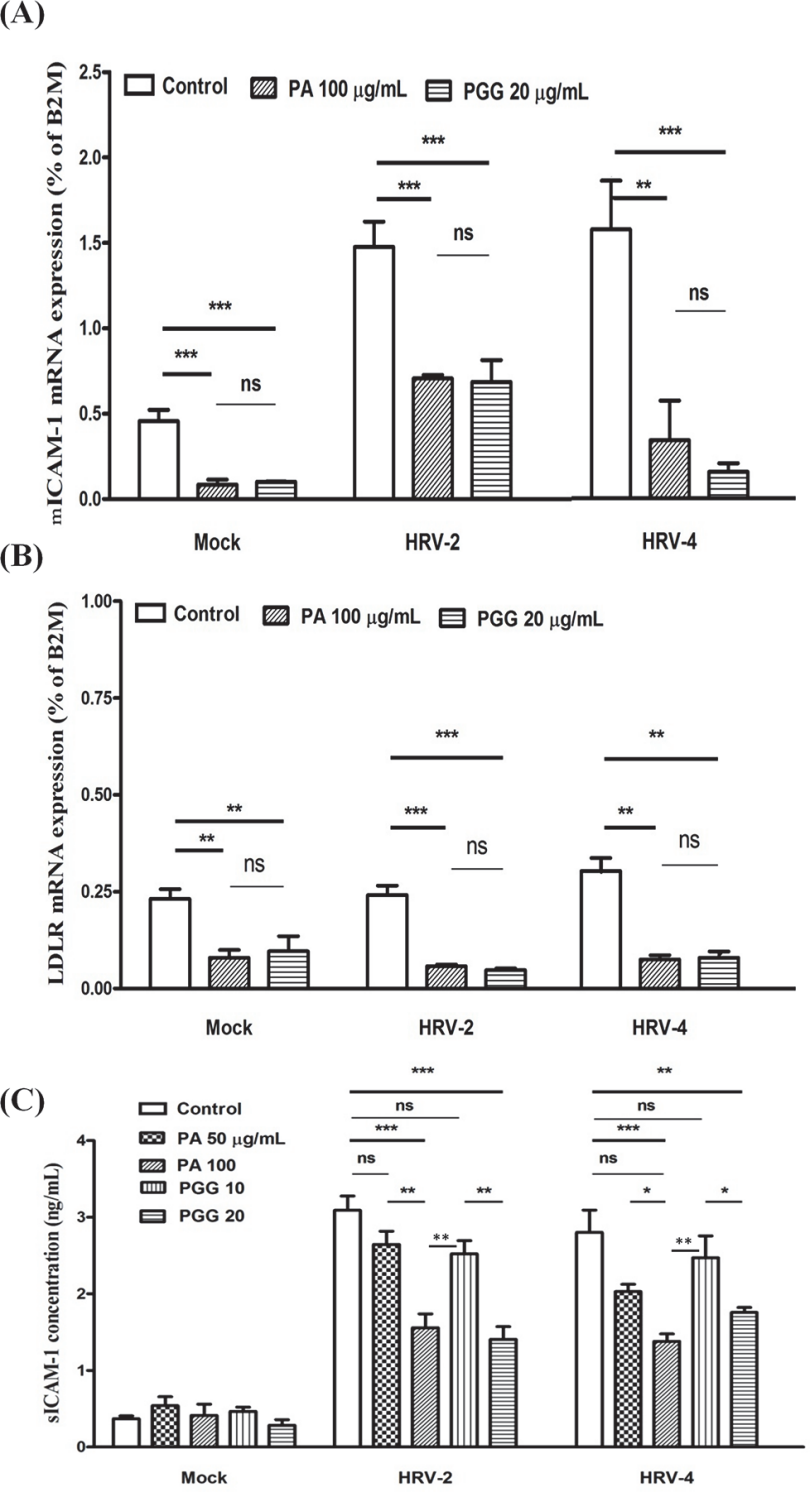
Effect on mRNA and protein expressions of ICAM1 and LDLR. The mRNA expressions of membrane-bound intercellular adhesion molecule-1 (mICAM-1) (**A**) and low-density lipoprotein receptor (LDLR) (**B**) were detected by real-time quantitative reverse transcription-PCR in MRC5 2 days after infection in the presence of 100 μg/mL paeonol (PA) and 20 μg/mL 1,2,3,4,6-penta-*O*-galloyl-β-D-glucopyranose (PGG). Soluble ICAM-1 (sICAM-1) (**C**) was detected by ELISA. Each bar represents the mean ± SD of triplicate samples of three independent experiments. ***p<0.001, **p<0.01, *p<0.05, using a Bonferroni multiple comparison post-test.

mRNA expression of ICAM-1 in MRC5 2 days after HRV infection in the presence of 100 μg/mL PA or 20 μg/mL PGG were investigated using real-time qRT-PCR (Fig 5A). HRV-2 or HRV-4 infection increased ICAM-1 mRNA expression, but these increases were reduced by the addition of PA and PGG. Furthermore, the ICAM-1 mRNA expression level in the group of PGG or PA treatment was lower than those in the group without the treatment with the test compounds. mRNA expression of LDLR in MRC5 was likewise assessed (Fig 5 B). LDLR mRNA expression in the HRV-2 or HRV-4 infected untreated cells was similar to that in the mock untreated cells and the expression decreased in the group of PA or PGG treatment. Using ELISA (Fig 5C), it was found that sICAM-1 levels in supernatants of HRV-2 or HRV-4 infected cultures were higher than those in supernatants of noninfected cultures, and sICAM-1 levels in supernatants of cultures treated with PA or PGG were significantly lower than those in cultures without the treatment with the test compounds. However, there was no difference in sICAm-1 levels between mock cultures with or without constituents.

**Fig 6:**
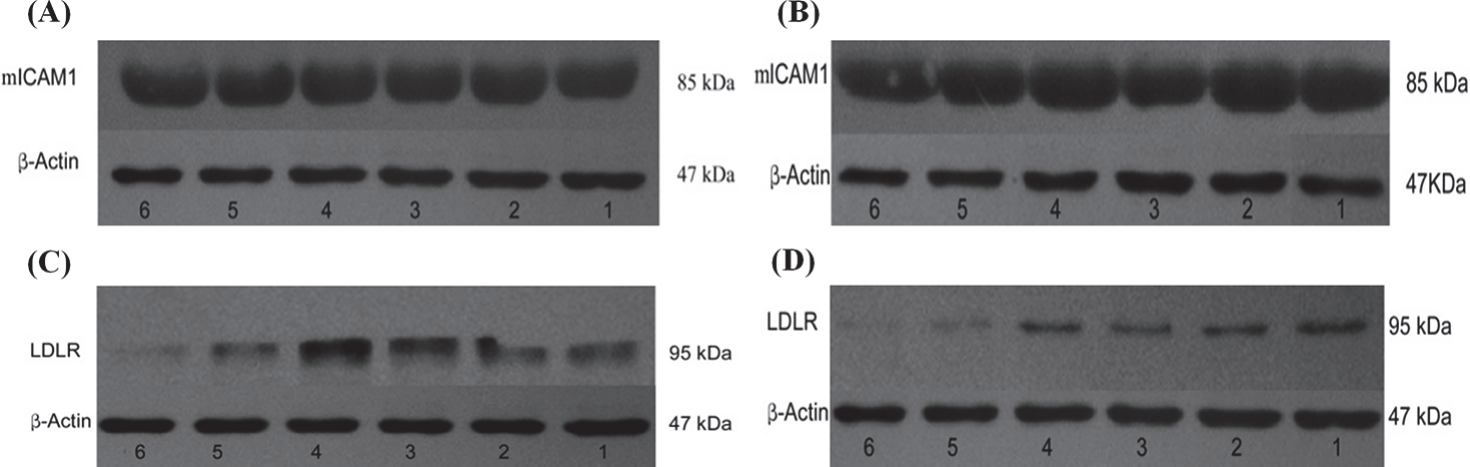
Western blot analysis of ICAM-1 and LDLR expressions. Intercellular adhesion molecule-1 (ICAM-1) and low-density lipoprotein receptor (LDLR) expressions in MRC5 cells 2 days after infection with HRV-2 (**A** and **C**) and HRV-4 (**B** and **D**) in the presence of paeonol (PA) or 1,2,3,4,6-penta-*O*-galloyl-β-D-glucopyranose (PGG). Lanes: (1) control (uninfected/untreated); (2) infected with HRV/untreated; (3) uninfected/treated with 100 μg/mL PA; (4) infected/treated with 100 μg/mL PA; (5) uninfected/treated with 20 μg/mL PGG; (6) infected/treated with 20 μg/mL PGG.

Western blot analysis of mICAM-1 and LDLR expressions in MRC5 cells 2 days after HRV infection in the presence of 100 μg/mL PA or 20 μg/mL PGG was performed. mICAM-1 protein levels in noninfected cultures were similar to those in cultures infected with HRV-2 or HRV-4, and the protein levels in cultures treated with PA or PGG at the test concentrations were also similar to those in cultures without treatment of the test compounds (Fig 6A for HRV-2; Fig 6B for HRV-4). The presence of 20 μg/mL PGG in cultures resulted in the sharp decline in the LDLR protein expression, compared with the cultures without PGG treatment, regardless of HRV-2 infection. However, 100 μg/mL PA had no effect on expression of LDLR protein (Fig 6C). Similar results were also observed with HRV-4 (Fig 6D).

### Effect on expression of cytokines

**Fig 7:**
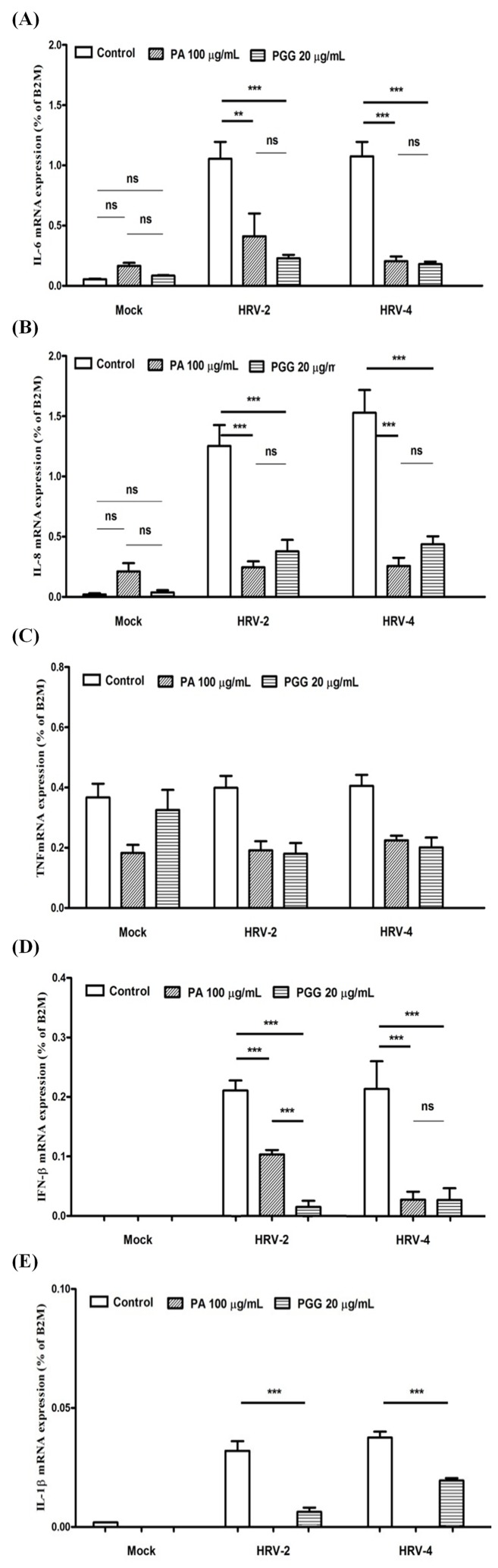
Effect on mRNA expressions of cytokines. mRNA expressions of interleukin (IL)-6 (**A**), IL-8 (**B**), tumor necrosis factor (TNF) (**C**), interferon beta (IFN-β) (**D**), and IL-1β (**E**) were detected by real-time quantitative reverse transcription-PCR in MRC5 2 days after infection in the presence of 100 μg/mL paeonol (PA) or 20 μg/mL 1,2,3,4,6-penta-*O*-galloyl-β-D-glucopyranose (PGG). Each bar represents the mean ± SD of triplicate samples of three independent experiments. ***p<0.001, **p<0.01, using a Bonferroni multiple comparison post-test.

mRNA expressions of various cytokines in MRC5 2 days after infection with HRV-2 or HRV-4 in the presence of 100 μg/mL PA or 20 μg/mL PGG were assessed using real-time qRT-PCR. HRV-2 and HRV-4 evoked a significant increase in IL-6 mRNA expression levels in the nontreated cell cultures, but the expression levels were significantly reduced in the cell cultures treated with PA or PGG, although there was no difference in IL-6 mRNA levels between mock cultures with or without constituents (Fig 7A). Similar results were also observed with IL-8 mRNA (Fig 7B). The expression of TNF mRNA in the culture challenged with HRV-2 or HRV-4 was similar to that in the noninfected cell cultures. The treatment with PA and PGG did not remarkably reduce the TNF mRNA expression, compared to the untreated cells (Fig 7C). Although the IFN-β mRNA expression did not occur in the noninfected cell cultures, IFN-β mRNA was expressed in the cell cultures infected with HRV-2 or HRV-4. The IFN-β mRNA expressions were meaningfully inhibited by PA or PGG treatment (Fig 7D). IL-1β mRNA expression was also highly induced by HRV-2 or HRV-4, and the induction disappeared or was significantly reduced by PA or PGG treatment (Fig 7E).

**Fig 8:**
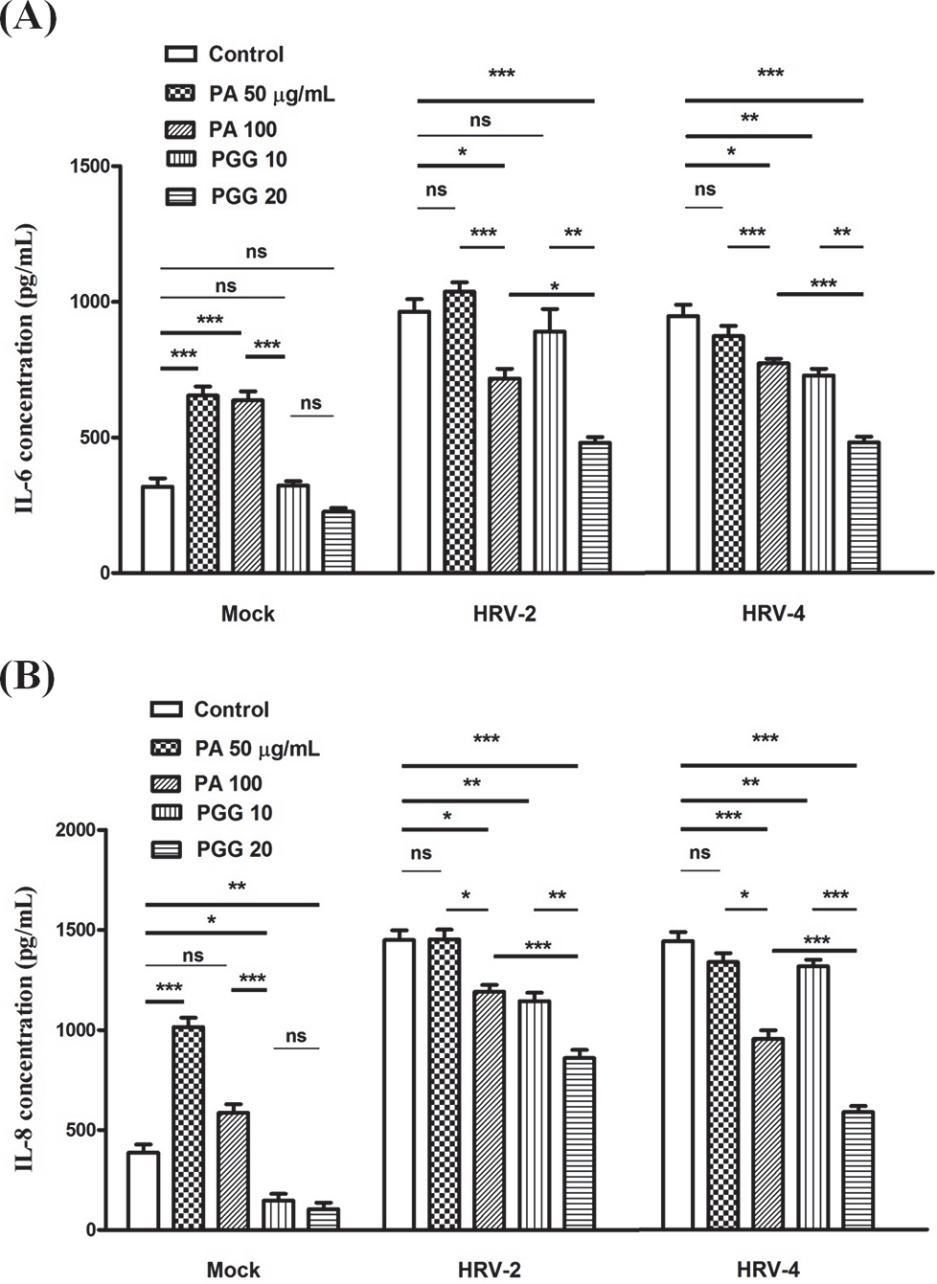
Effect on protein expression of cytokines. Concentrations of interleukin (IL)-6 (**A**) and IL-8 (**B**) was detected by ELISA in MRC5 supernatants after 2 day infection in the presence of 100 μg/mL paeonol (PA) and 20 μg/mL 1,2,3,4,6-penta-*O*-galloyl-β-D-glucopyranose (PGG). Each bar represents the mean ± SD of triplicate samples of three independent experiments. ***p<0.001, **p<0.01, using a Bonferroni multiple comparison post-test.

The concentrations of IL-6 detected by ELISA in the HRV-2 or HRV-4 infected cell supernatants were significantly higher than those in the noninfected cell supernatants. IL-6 concentrations in the mock cultures treated with PA were higher than those in the mock cultures without the treatment. However, IL-6 concentrations in infected cultures were reduced by treatment with 100 μg/mL PA or 20 μg/mL PGG. The decrease in HRV-induced IL-6 secretion by 20 μg/mL PGG treatment was higher than that by 100 μg/mL PA treatment. PA and PGG affected IL-6 protein expressions in a concentration-dependent manner (Fig 8A). Similar results were observed with IL-8 (Fig 8B). However, TNF protein was not detectable in supernatants of all the cultures (Data not shown).

### Effect on TLR3 mRNA expression

**Fig 9:**
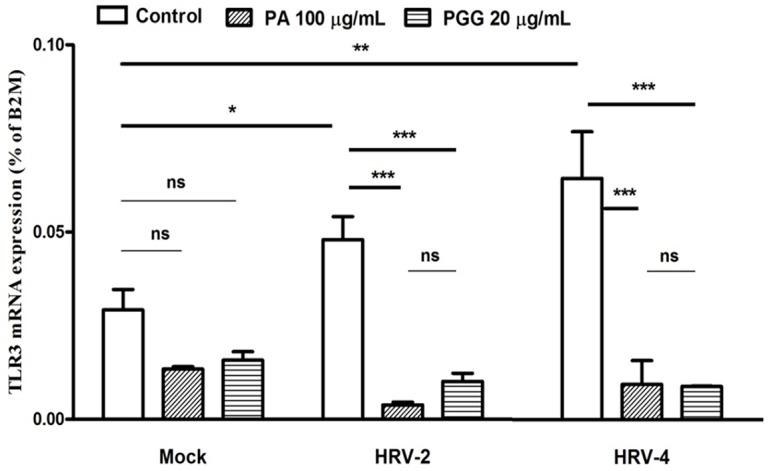
Effect on expression of TLR3 mRNA. Toll-like receptor 3 (TLR3) mRNA expression was detected by real-time quantitative reverse transcription-PCR in MRC5 2 days after infection in the presence of 100 μg/mL paeonol (PA) and 20 μg/mL 1,2,3,4,6-penta-*O*-galloyl-β-D-glucopyranose (PGG). Each bar represents the mean ± SD of triplicate samples of three independent experiments. ***p<0.001, **p<0.01, using a Bonferroni multiple comparison post-test.

TLR3 mRNA expression in HRV-2 or HRV-4 infected cells was increased, compared with uninfected cells (Fig 9). TLR3 mRNA expression in the cultures treated with 100 μg/mL PA or 20 μg/mL PGG was significantly lower than that in the cultures without the treatments with the test compounds.

## Discussion

Many plants and their constituents such as phenolics, terpenoids and alkaloids manifest antiviral activity toward different viruses [12,25] and have been proposed as alternatives to conventional antiviral drugs. Anti-HRV constituents derived from plants include alkaloids (e.g., arborinine and (*S*)-ribalinine, IC_50_ 3.19 and 82.95 μM [26]; glaucine, IC_50_ 22 μM [27]), coumarins (e.g., 6,7,8-trimethoxycoumarin and daphnoretin methyl ether, IC_50_ 11.98 and 97.08 μM [26]; farnesiferol B and C, IC_50_ ≈2.61 μM [28]), flavonoids (e.g., 4ʹ,5-dihydroxy-3ʹ,3,7-trimethoxyflavone, IC_50_ 0.29 μM [29]; 3-methylquercetin and three related compounds, effective concentration 15.8 μM [30]; chrysosplenol D and others, minimum effective dose 0.22–33.3 μM [31]), terpenoids (e.g., 3-*O*-*trans*-caffeoyltormentic acid, IC_50_ 30.72 μM [32]; orobol 7-*O*-D-glucoside, IC_50_ 1.29–19.62 μM [33]), and organic acid (e.g., raoulic acid, IC_50_ 0.51 μM [34]; gallic acid, IC_50_ ≈294.55 μM [35]). It has been reported that HRV capsid-binding compounds toward all HRV serotypes showed the existence of group A and B, based on a wide range of susceptibilities to antiviral compounds [36].

In the current study, the antiviral principles of *P*. *lactiflora* root was determined to be the aryl ketone PA (**1**), the simple benzoic acid GA (**2**), and the *gluco*-hexose PGG (**3**). These constituents exhibited antiviral activity toward both group A (HRV-2) and group B (HRV-4). IC_50_ of PGG, GA, and PA toward two HRVs was between 11.56 and 17.89 μM, between 426.99 and 448.10 μM, and between 492.17 and 608.38 μM, respectively, although IC_50_ of the natural compounds stated previously is between 0.22 and 294.55 μM. PGG exhibited greater antiviral activity than ribavirin (IC_50_, 240.49–324.07 μM) toward two HRVs and high selectivity. The virus titration assay results also proved that the constituents had antiviral activity toward the HRVs. This original finding indicates that materials derived from *P*. *lactiflora* root can hold promise for the development of novel and effective naturally occurring antiviral agents for two different HRV groups A and B. PGG was reported to possess antiviral activity toward hepatitis B virus [37] and influenza A virus [38].

Investigations on the modes of action of natural antivirals may contribute to the development of selective HRV therapeutic alternatives with novel target sites. The modes of antiviral action of plant secondary substances have been well documented by Rollinger and Schmidtke [1] and Kitazato et al. [39]. Targeting virus molecules is likely more specific and less toxic, but there is a narrow spectrum of viruses and a higher risk of creating resistant viruses [39]. On the contrary, chemicals which target cellular molecules may possess a broader antiviral activity spectrum and less risk of developing virus resistance, but may be more toxic to the host cell [39]. In the current study, PGG and PA do not interact with the HRV-4 particles, as preexposure of the virus to the constituents did not alter the infectivity of HRV-4 particles. Based on time-course tests, PGG and PA can inhibit HRV-4 infection only when they were added during the virus inoculation (0 h), but not after 1 h or later. This finding suggests that PGG and PA may interact with the human cells in the early stage of HRV infections to protect the cells from the virus destruction. The mechanism of action of these constituents on cellular protection toward HRV-4 still remains unclear, although ribavirin was reported to enter host cells through nucleoside transporters in several studies [40,41]. In addition, real-time RT-PCR analysis revealed that the RNA replication levels of HRVs were remarkably reduced in the MRC5 cultures treated with these constituents. This finding suggests that PGG and PA may block or reduce the entry of the viruses into the cells in the early stage of HRV infections to protect the cells from the virus destruction and abate virus replication. Detailed tests are needed to fully understand the modes of action of the constituents. It has been suggested that the mode of anti-HRV action of gallic acid might be derived from the inhibition of virus absorption [35].

Interference with specific virus-host cell receptor interactions can be one of the potential interventions in antiviral chemotherapy [42]. HRVs are classified into the major and minor groups, based on their binding to ICAM-1 [43,44] or to members of the LDLR family [45], respectively. ICAM-1 is a critical target-docking molecule on epithelial cells for 90% of the HRV serotypes [43,44]. The major group uses ICAM-1 as a mechanism to gain entry to the host cell [46]. It has been suggested that antagonism of the virus-receptor interaction would appear to be an effective way to inhibit a broad spectrum of HRVs [47]. Macrolide antibiotics such as erythromycin inhibit infection by the major group of HRVs via a reduction in ICAM-1 expression [48]. mICAM-1 expression is equally induced by both major and minor group HRVs [49]. The HRV-induced ICAM-1 up-regulation is not receptor-restricted [49]. Moreover, it has been reported that pretreatment of HRV-2 with sICAM did not alter the ability of HRV-2 to induce ICAM-1 [46]. The current experiments demonstrated that both HRV-2 and HRV-4 induced the both forms of ICAM-1 expressed in MRC5. ELISA results revealed that sICAM-1 protein levels of infected cultures treated with PA or PGG were significantly lower than those in cultures without the compound treatment, although there was no difference in sICAm-1 levels between mock cultures with or without the constituents. However, ICAM-1 mRNA expression levels were decreased in the presence of PGG or PA in both infected and noninfected cell cultures. The findings indicated that these constituents interfered with host cell protein expression of this receptor through inhibition of the receptor RNA expression or reduction of HRV replication. In addition, LDLR mRNA expressions were also reduced in the presence of PGG and PA in the cultures. However, LDLR protein expression was inhibited in PGG treatment only. The findings suggest that PGG interfere with LDLR expression through inhibiting expression of LDLR RNA or reduction of HRV replication, whereas PA interfered with LDLR expression through only reduction of HRV replication. PA was reported to suppress ICAM-1 expression in TNF-stimulated human umbilical vein endothelial cells [50].

HRV infection induces the production of numerous components of innate immunity including TLR3 and imflamatory mediators, such as kinins, leukotrienes, histamine, interleukins (IL-1, IL-6, and IL-8), TNF, and regulated by activation normal T cell expressed and secreted [2,51]. The surface-expressed TLR3s were reported to have an important function in response to a common human viral infection of natural host cells and play an important role in innate immune responses toward HRV infection [52,53]. During the replication cycle of HRV, viral double-stranded RNA (dsRNA) is recognized by TLR3 [2]. The interaction between dsRNA and TLR3 activates a signaling cascade that triggers increase of cytokine production in host cells and stimulates innate immune responses [2]. It was supposed that inhibition of TLR3 expression have potential modulatory effects on the pathophysiologic changes related to HRV infection [53]. Cytokines such as TNF, IL-1β, IL-6, and IL-8 induce upregulation expression of the adhesion molecules such as ICAM-1 in both epithelial and vascular endothelial cells, thereby increasing cell susceptibility to HRV infection [54]. The concentrations of IL-6 and IL-8 in nasal secretions correlate with the severity of the symptoms in patients with colds [55]. HRV infections were reported to be responsible for triggering exacerbations of asthma through inducing gene expression of these cytokines in asthmatic subjects [56]. ELISA results revealed that PA induced IL-6 and IL-8 expression. Therefore, the expression levels in the infected cell cultures treated with PA were lower than those in the infected cell cultures without the constituent as a consequence of the HRV replication reductions. PGG reduced IL-6 and IL-8 expression and the decreases of expression levels in the infected cell cultures treated with PGG were resulted from the interference of the constituent with both of host cell protein expression and HRV replication. The current study also indicated that the constituents reduced expressions of TLR3 and cytokines, such as TNF, IFN-β, and IL-1β, which resulted in diminishing symptoms induced by HRV. It may be that effects of the constituents on blocking or reducing the entry of the viruses into the cells, which results in reducing HRV-RNA replications, play an important role in interfering with HRV-induced gene expression.

In conclusion, *P*. *lactiflora* root-derived preparations containing PGG and PA could be useful as an antiviral agent in the prevention or treatment of HRV infection. The antiviral action of PGG and PA may be an indication of at least one of the pharmacological actions of *P*. *lactiflora*. For the practical use of the preparations as novel anti-HRV products to proceed, further research is needed to establish their safety with respect to humans and whether this activity is exerted *in vivo* after consumption of *P*. *lactiflora* root-derived products by humans. Lastly, detailed tests are needed to understand how to improve anti-HRV potency and stability for eventual commercial development.
